# Expression and clinical significance of Klotho protein in serum, umbilical cord blood, and placenta of pregnant women with intrauterine growth restriction

**DOI:** 10.3389/fped.2025.1611877

**Published:** 2025-08-04

**Authors:** Guixiang Zeng, Jingjing Lian, Jiajia Shen, Yuan Shi

**Affiliations:** ^1^ Chongqing Key Laboratory of Pediatrics, Ministry of Education Key Laboratory of Child Development and Disorders, Department of Neonatology, National Clinical Research Center for Child Health and Disorders, Children's Hospital of Chongqing Medical University, Chongqing, China; ^2^ Department of Neonatology, Nanning Maternity and Child Health Hospital, Nanning, China

**Keywords:** Klotho, intrauterine growth restriction, placenta, growth hormone, insulin-like growth factor-1

## Abstract

**Objectives:**

Intrauterine growth restriction (IUGR) significantly affects neonatal development, but its pathogenesis is not fully understood. Klotho protein is involved in aging-related diseases, and its role in fetal growth is unclear. This study aims to explore Klotho's role in IUGR.

**Methods:**

A case-control study was conducted at Nanning Maternal and Child Health Care Hospital from July 2023 to June 2024. Fifty-two neonates (gestational age ≥34 weeks and <42 weeks) were divided into the appropriate for gestational age (AGA) group (*n* = 30) and the IUGR group (*n* = 22). Venous and umbilical cord blood were collected to measure Klotho, growth hormone (GH), and insulin-like growth factor 1 (IGF-1). Placental tissues were examined for histopathology and immunohistochemistry.

**Results:**

The IUGR group showed placental morphological changes, including increased syncytial knots and inflammation. Klotho expression in placental tissue was significantly reduced (*p* < 0.0001), while IGF-1 levels increased (*p* < 0.001) and GH levels decreased (*p* < 0.001). Soluble α-Klotho levels were lower in maternal venous blood (*p* < 0.0001) and umbilical cord blood (*p* < 0.01). GH and IGF-1 levels in maternal venous blood and umbilical cord blood were altered in IUGR cases.

**Conclusions:**

Reduced Klotho expression in IUGR cases, along with changes in GH and IGF-1, suggests disruptions in metabolic processes affecting fetal growth and development. These findings suggest a potential involvement of Klotho in placental changes and fetal development, warranting further mechanistic studies.

## 1 Introduction

Intrauterine growth restriction (IUGR) is a condition where the fetal weight is below the 10th percentile for gestational age. Globally, IUGR affects approximately 5% to 10% of pregnancies (1). The etiology of IUGR is multifactorial and is primarily attributed to alterations in placental function. In addition to maternal complications during pregnancy, intrauterine infections, and nutritional deficiencies, studies have also investigated genetic changes in the placenta and modifications in fetal endocrine hormones. Risk factors for IUGR are classified into maternal, placental, and fetal components, and are notably influenced by endocrine metabolism (2). Klotho proteins are primarily single-pass transmembrane proteins, consisting of secreted α-Klotho (α-KL or sKL) and membrane-bound β-Klotho (β-KL or mKL).Its function is complex and involves multiple signaling pathways. Membrane-bound Klotho, as a co-receptor for fibroblast growth factors 23 (FGFs 23), participates in the regulation of renal phosphate excretion. In addition, the function of secreted Klotho involves the regulation of several cell surface glycoproteins, including ion channels and growth factor receptors (3, 4). Recent studies have demonstrated that α-KL plays a key role in the endocrine regulation of infant mineral metabolism and postnatal growth, as well as in essential biological processes such as antioxidant enzyme activity, nitric oxide production, and angiogenesis (5–8). β-Klotho is expressed in various tissues, including the liver, exocrine pancreas, adipose tissue, and brain. It regulates lipid metabolism, glucose metabolism, and energy homeostasis through activation of the Fibroblast Growth Factor (FGF) 15 or FGF21 signaling pathways (9–13). This pathway may contribute to muscle development in IUGR (14). The growth hormone (GH)/insulin-like growth factor (IGF)-1 axis is vital for many metabolic functions, including proper development and growth of bones, skeletal muscles, and adipose tissue. Defects in the axis' activity during childhood result in growth abnormalities, while increased secretion of GH from the pituitary results in acromegaly. So as to keep normal physiologic concentration, the secretion and activity of GH and IGF-1 are closely regulated by hypothalamic, pituitary, endocrine, paracrine, and autocrine factors (15, 20). The positive correlation between α-Klotho and neonatal body weight and length may reflect its role in promoting growth, similar to that of IGF-1. Although it is currently unclear whether this association is directly mediated by α-Klotho or through the influence on IGF-1 (5, 7). This study aims to investigate the differential expression of Klotho protein as a biomarker between pregnant women with and without IUGR and its impact on the GH/IGF-1 axis in fetal development. Ultimately, this study aims to identify an early biomarker for the onset and progression of IUGR and assess the feasibility of intrauterine interventions.

## 2 Materials and methods

### 2.1 Subjects

From July 2023 to June 2024, pregnant women and their newborns admitted to the Nanning Maternal and Child Health Hospital of Guangxi were recruited. The inclusion criteria were as follows: (1) Gestational age between 34 + 0 and 41 + 6 weeks (singleton pregnancy confirmed by early ultrasound); (2) Birth weight ≦90th percentile for gender and gestational age(using reference standards and growth curves for neonatal birth weight at various gestational ages and sexes by Zhu Li, Zhang Rong); (3) Availability of complete prenatal growth monitoring data (≥3 ultrasound records); (4) Signed informed consent from both pregnant women and their families. The exclusion criteria were: (1) Fetal conditions: major congenital anomalies (ICD-10 codes Q00-Q99), chromosomal abnormalities, stillbirth or termination of pregnancy cases; (2) Maternal conditions: critical obstetric conditions (amniotic fluid embolism, uterine rupture, sepsis, et al), multiple pregnancies, gestational age <34 weeks, infectious diseases(HIV,HBV,syphilis, et al); (3) Incomplete prenatal examination information and materials; (4) Discrepancy >2 weeks between LMP and early ultrasound dating; (5) Participant withdrawal at any stage Participants were divided into two groups: IUGR group and AGA group. This study was approved by the Ethics Committee of the Nanning Maternal and Child Health Hospital, and written informed consent was obtained from all participants or their families prior to enrollment.

### 2.2 Diagnostic criteria for IUGR and appropriate for gestational age (AGA)

Reference standards and growth curves for neonatal birth weight at various gestational ages and sexes were reported by Zhu Li, Zhang Rong, and colleagues in 2015 (16). Intrauterine growth restriction (IUGR) was defined according to the following criteria: (1) Birth weight below the 10th percentile for the corresponding sex and gestational age (BW < 10th percentile); (2) First-trimester ultrasound assessment to confirm gestational age and evaluate fetal morphology;(3) Unlike SGA, IUGR focuses on the fact that a fetus fails to reach its genetic potential due to pathological factors such as placental insufficiency, infection, maternal disease, essentially pathological growth disorder, SGA encompasses pathological IUGR as well as physiological low birthweight infants. The diagnostic criteria for appropriate-for-gestational-age (AGA) neonates were as follows: (1) Birth weight within the 10th to 90th percentiles for the corresponding sex and gestational age; (2) Confirmation of gestational age through early pregnancy ultrasound examination.

### 2.3 Hematoxylin and eosin staining

The placental tissue was frozen, sectioned into 6 µm sections and stained with HE staining kit (Solarbio, #G1120). Specifically, the 6 µm thick sections of the placenta were air-dried at room temperature for 10–15 min and then stained with hematoxylin solution for 5 min, washed with distilled water to remove excess stain, dipped in differentiating solution for 3–5 s, and then rinsed with water twice for 3–5 min each time. The sections were then stained with eosin solution for 30 s to 2 min, excess stain was removed, and the sections were rapidly dehydrated. The slides were sequentially immersed in ethanol gradients of 75%, 85%, 95%, and 100% for 2 to 3 s each. The slides were immersed in 100% ethanol for 1 min and subsequently cleared with xylene twice, each for 1 min, and mounted using neutral balsam mounting medium (Shenggong Bioengineering Shanghai Co. LTD, #F418FD0271). A total of 5 biological samples were analyzed per group,The placental tissue was then assessed by scoring the H&E-stained sections under 200 ×  magnification as reported previously.

### 2.4 Immunohistochemistry

The samples were soaked in a 3% hydrogen peroxide solution at room temperature for 15 min to eliminate endogenous catalase. The tissue sections were then immersed in phosphate-buffered saline (PBS) three times, with each soak lasting 3 min. The samples were treated with 5% normal goat serum for 1 h at room temperature to block nonspecific antigens. The excess serum was removed without washing. The sections were incubated with a blocking solution at a 1:500 dilution for 90 min at room temperature. The samples were subsequently r rinsed with PBS three times, for 5 min each. Next, the slides were incubated with an HRP-conjugated secondary antibody at a 1:70 dilution for 60 min. The samples were subsequently collected and washed with PBS three times, each for 5 min. DAB was then applied to the slides until the chromogenic reaction was visible, resulting in a brown color under microscopic observation, typically within 1–10 min. The slides were washed with distilled water twice, each for a duration of 5 min. Following this, the sections were stained with hematoxylin for 5 min. If excess dye was observed, the slides were rinsed with 1% hydrochloric acid ethanol for about 5 s, followed by a rinse in tap water until the stain turned blue. The sections were dehydrated with a gradient series of alcohols at 70%, 80%, 90%, 95%, and 100% concentration for 3 min each. The slides were then cleared twice with xylene for 5 min. Neutral balsam was used to seal the sections, and photographs were taken. The antibodies used in this study included a primary antibody against Klotho (Rabbit anti-Human/Mouse/Rat, Proteintech, Cat# 28100-1-AP) and an HRP-conjugated secondary antibody [Goat anti-Rabbit IgG (H + L), ZSGB-Bio, Cat# SP-9001], both of which were used for immunohistochemical analysis. IHC-stained sections were imaged using a light microscope at ×200 magnification. A total of 10 biological samples were analyzed per group. Quantification of IHC staining was performed using ImageJ software. Ten randomly selected fields per section at ×200 magnification were analyzed to calculate mean optical density (MOD), which served as the indicator of expression level.

### 2.5 Immunofluorescence

The tissue specimens were air-dried at room temperature, then immersed in anhydrous methanol (DAMAO, #67-56-1) at −20°C for 10 min. Following this, they were rinsed three times with PBS, each wash lasting 5 min, dried completely, and outlined with a hydrophobic barrier pen to prevent overflow. The slides were blocked with PBS containing 1% BSA at room temperature for 40 min to block nonspecific binding. They were then incubated with primary antibodies overnight. After incubation, the samples were rinsed three times with PBS. Subsequently, they were incubated with secondary antibodies at room temperature for 1 h and washed three times with PBS in the dark. Finally, the placenta samples were mounted with coverslips using Anti-Fade Mounting Medium (Beyotime, #P0126-25 ml).Immunofluorescence images were acquired using a fluorescence microscope at ×200 magnification. Quantification of positive staining was done using ImageJ, calculating fluorescence intensity across 5 randomly selected fields.

### 2.6 RT–qPCR

RNA was extracted from the maternal placenta, and the concentration was assessed using a Nanodrop 2000 (Thermo, USA). cDNA synthesis was performed using reverse transcription with the PrimeScript™ RT reagent Kit with gDNA Eraser (Perfect Real Time) from TaKaRa. The reverse transcription conditions included 37 ℃ for 15 min, 85 °C for 5 s, and completion of the reactions on ice. qPCR was performed using the PerfectStart® Green qPCR SuperMix Kit with the qTOWER 3G Real-Time PCR System (Analytik Jena AG, Germany) according to the manufacturer's instructions. The primers used for Klotho were designed as follows: Klotho-F: 5′-ATGCCGAGCAAGACTCACTGA-3′ and Klotho-R: 5′-ACGCAAAGTAGCCACAAAGGT-3′. For GAPDH, the primers included GAPDH-F: 5′-AGGTCGGTGTGAACGGATTTG-3′ and GAPDH-R: 5′-TGTAGACCATGTAGTTGAGGTCA-3′. All of these primers were obtained from Sangon Biotech Ltd., located in Shanghai, China. We have specified that 200–1,000 ng of total RNA was used per RT-qPCR reaction, depending on sample concentration. Three biological replicates were analyzed per group (*n* = 3). Performed using SYBR Green. Relative gene expression was calculated using the 2^-*ΔΔ*Ct method, normalized to GAPDH. Each reaction was run in technical duplicate or triplicate.

### 2.7 ELISA

Maternal venous and umbilical cord blood were collected and centrifuged at 3,500 rpm for 10–15 min. The collected supernatant was transferred to a new centrifuge tube and stored at −20 °C or −80 °C until further use. Human serum and umbilical cord blood concentrations of GH, IGF-1, and IGFBP-1 were measured using ELISA kits specific to human targets: GH (Abbexa, Cat# abx250478), IGF-1 (Abcam, Cat# ab100545), and IGFBP-1 (Thermo Fisher Scientific, Cat# EHIGFBP1), All procedures were performed strictly according to the manufacturers' instructions. Following the kit instructions, serial dilutions of Klotho standards, typically from high to low concentrations, are prepared in 6–8 different concentrations. For example, 0 pg/ml, 50 pg/ml, 100 pg/ml, 200 pg/ml, 400 pg/ml, 800 pg/ml, and 1,600 pg/ml. Each concentration is tested in duplicate or triplicate. Different concentrations of standard solutions were added to the corresponding wells of the microplate, with 100 μl typically added to each well. The prepared samples were added to the wells of the microplate, with 100 μl added to each well. Similarly, each sample was tested in duplicate or triplicate. After sample loading, the microplate was sealed with a plate sealing membrane and incubated in an incubator maintained at 37 °C for 1–2 h to allow the Klotho antigen in the sample to bind to the antibody coated on the microplate. At the end of incubation, the microplate was removed, and the liquid in the wells was discarded, followed by washing with a wash buffer. Generally, the wells were washed 3–5 times, with 300–400 μl of wash buffer added to each well each time. After soaking for 1–2 min, the liquid in the wells was blotted dry. 100 μl of biotin-conjugated anti-Klotho detection antibody was added to each well, and the microplate was sealed with a plate sealing membrane and incubated in an incubator maintained at 37 °C for 1 h. The microplate was washed 3–5 times with wash buffer to remove unbound assay antibodies. 100 μl of enzyme-labeled avidin was added to each well, and plates were sealed and incubated at 37°C for 30–60 min. The washing operation was repeated to ensure adequate removal of unbound enzyme-labeled avidin. 100μl substrate solution was added to each well, gently mixed, and then the microplate was placed into a constant temperature incubator at 37 °C for 15–30 min in the dark for color development, and the liquid color change in the well was observed. When the appropriate degree of color development was reached, 50 μl of termination solution was added to each well to terminate the enzymatic reaction, at which point the color no longer changed. The microplate was placed into the microplate reader and the appropriate wavelength (usually 450 nm) was selected to read the absorbance value (OD) of each well.

### 2.8 Masson stain

The 6 µm-thick slide sample was carefully removed and air-dried at room temperature for 10–15 min. Subsequently, the cells were stained with freshly prepared Weigert iron hematoxylin for 5–10 min, followed by differentiation using an acidic ethanol solution. The samples were then rinsed thoroughly with distilled water, treated with Masson's bluing solution to restore blue coloration, and washed again with distilled water. Ponceau fuchsin staining solution was applied for 5–10 min. During this process, a weak acid working solution (prepared in a ratio of distilled water to weak acid solution = 2:1) was used to wash the samples for 1 min. The samples were further washed with phosphomolybdic acid solution for 1–2 min, followed by another wash with the weak acid working solution for 1 min. Aniline blue staining solution was then applied for 1–2 min, followed by an additional wash with the weak acid working solution for 1 min. Finally, the slides were rapidly dehydrated with 95% ethanol, dehydrated in three cycles with absolute ethanol (each lasting 5–10 s), cleared with xylene in three cycles (each lasting 1–2 min), and mounted with neutral resin. Five biological replicates were analyzed per group (*n* = 5). Masson's trichrome-stained sections were observed and photographed using an optical microscope at ×200 magnification.

### 2.9 Statistical analysis

All data were plotted and subjected to statistical analysis using GraphPad Prism version 10.1.2. An unpaired *t*-test with Welch's correction was employed to compare the means between two independent samples. For comparisons involving multiple groups, one-way ANOVA was used. Measurement data are presented as mean ± standard deviation (mean ± SD). Statistical significance was defined as two-tailed *p* < 0.05.

## 3 Results

### 3.1 Clinical characteristics of the study population

Fifty-two subjects who met the inclusion criteria were recruited from Nanning Maternal and Child Health Hospital. The subjects' basic demographic characteristics are shown in Table 1. The subjects were divided into two groups: AGA groups (*n* = 30) and IUGR groups (*n* = 22). The mea*n* ± standard deviation of the age of the pregnant women in the AGA group was 30.53 ± 5.29 years (range 20–40 years), and that in the IUGR group was 31.04 ± 4.57 years (range 22–38 years). Statistical analysis was performed using an unpaired *t*-test for continuous variables and a chi-square test for categorical variables, it revealed that the gestational age at delivery and birth weight in the IUGR group were significantly lower than those in the AGA group (****p < 0.0001) (Table 1).

**Table 1:** General characteristics of the study population.

Characteristic	^c^AGA (*n* = 30)	^d^IUGR (*n* = 22)	*p* value
Maternal age (Years) ($\bar{\chi }$ ± s)	30.53 ± 5.29	31.04 ± 4.57	0.7138
Pregnancy Number ($\bar{\chi }$ ± s)	2.70 ± 7.51	2.04 ± 1.15	0.0891
Number of births ($\bar{\chi }$ ± s)	1.66 ± 0.76	1.39 ± 0.58	0.1236
^a^GA at Delivery(weeks) ($\bar{\chi }$ ± s)	38.80 ± 0.85	34.40 ± 4.20	****p < 0.0001
Birth Weight (grams) ($\bar{\chi }$ ± s)	3,242 ± 379.2	2,253 ± 381.6	****p < 0.0001
Height of pregnant (cm) ($\bar{\chi }$ ± s)	157.0 ± 4.35	154.9 ± 5.71	0.1298
^b^BMI of pregnant (kg/m^2^) ($\bar{\chi }$ ± s)	22.39 ± 3.98	21.44 ± 4.10	0.4217
Maternal weight gain (kg) ($\bar{\chi }$ ± s)	14.03 ± 5.37	14.98 ± 2.64	0.4571

^a^ GA (Gestational age).

^b^ BMI (Body Mass Index).

^c^ AGA (appropriate for gestational age).

^d^ IUGR (Intrauterine Growth Retardation).

**** *P* < 0.0001, statistical significance.

### 3.2 Abnormal expression of Klotho protein in IUGR

#### 3.2.1 HE staining was used to assess the morphological heterogeneity of IUGR placentas

**Figure 1:**
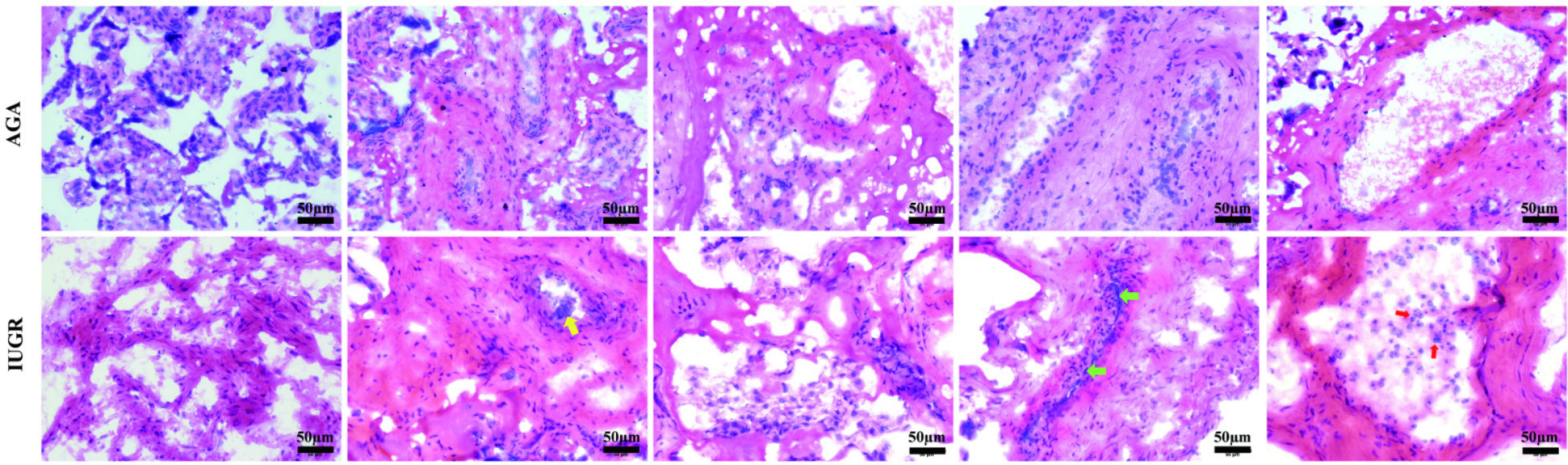
HE staining of the AGA group and IUGR group. Representative HE staining of the AGA placental tissue shows intact morphology. HE staining of the IUGR tissue reveals that, compared with the AGA group, the morphology of the IUGR tissue has changed, with syncytial knots (yellow arrows), diffuse inflammation (red arrows), and calcification (green arrows). The scale in the figure is 50 μm, magnification: 200× times.

To assess the pathological changes in placental tissue and validate the clinical diagnosis, placental tissue samples were collected from two groups of pregnant women. We collected placental tissue from the central area of the fetal side of the placenta, approximately 5 cm in diameter, and classified placental abnormalities including maternal vascular malperfusion (MVM), fetal vascular malperfusion (FVM), villitis of unknown etiology (VUE), and acute chorioamnionitis according to the Amsterdam Placental Workshop Group Consensus (2016) criteria (17). The tissues were embedded in FSC 22 Clear Frozen Section Compound, frozen using liquid nitrogen, and sectioned into 6 µm thick sections for hematoxylin and eosin (HE) staining. As illustrated in Figure 1 (magnification: 200× times.), the blood vessels in the AGA group were intact, with normal syncytiotrophoblasts covering the placental villi. In contrast, the sections from the intrauterine growth restriction (IUGR) group showed distinct morphological alterations, including a higher number of syncytial knots (indicated by yellow arrows), extensive diffuse inflammation (marked by red arrows), and vascular calcification (highlighted by green arrows).

#### 3.2.2 The expression levels of soluble α-Klotho in both venous blood and umbilical cord blood from IUGR women were decreased. Additionally, the expression of Klotho mRNA in the placenta was decreased

**Figure 2:**
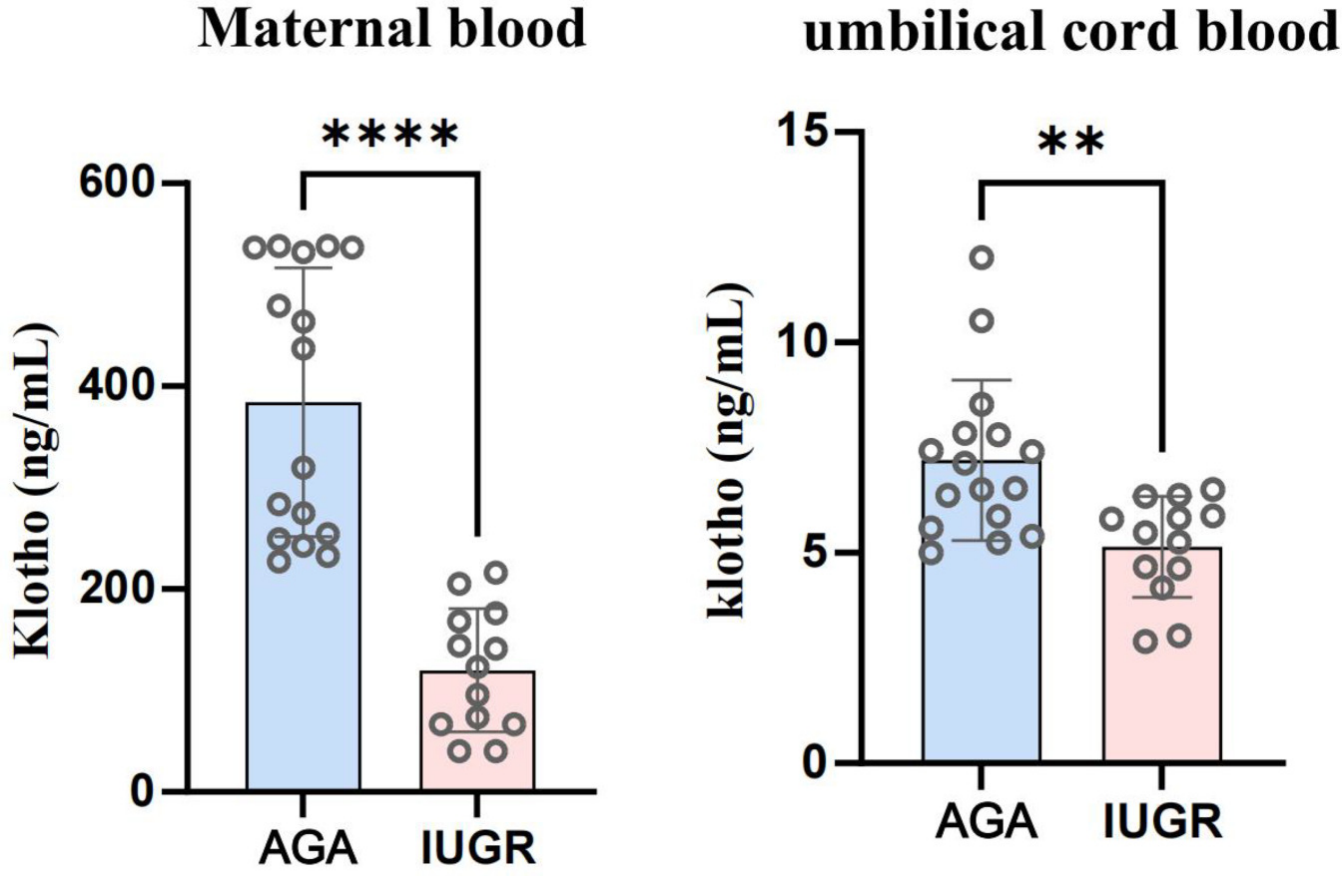
Soluble α-Klotho levels in venous blood and umbilical cord blood of IUGR pregnant women were significantly lower than those in the AGA group (****: *p* < 0.0001, ***: *p* < 0.001, **: *p* < 0.01, *: *p* < 0.05, ns, not statistically significant).

**Figure 3:**
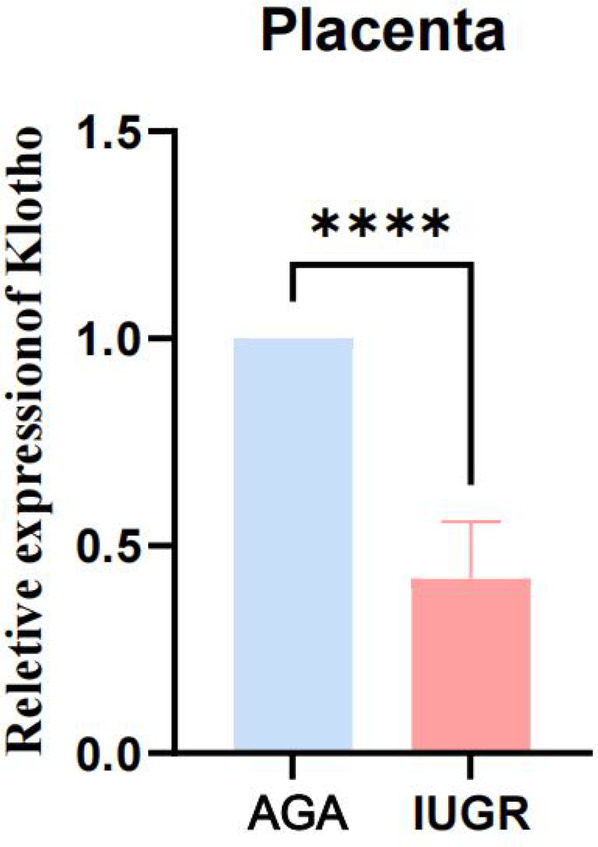
The expression of Klotho mRNA in IUGR placentas is significantly lower than that in the AGA group. (****: *p* < 0.0001, ***: *p* < 0.001, **: *p* < 0.01, *: *p* < 0.05, ns: not statistically significant).

We collected venous and umbilical cord blood samples from pregnant women in both the AGA group and the IUGR group. Serum was isolated by centrifugation at 3,500 rpm. The concentration of soluble α-Klotho in maternal blood and umbilical cord blood was measured using a Kl-specific ELISA kit for both groups. An unpaired *t*-test with Welch's correction was employed to compare the meanings between two independent samples. For comparisons involving multiple groups. The results demonstrated that the concentration of soluble α-Klotho in maternal blood and umbilical cord blood of the IUGR group was significantly lower than that in the AGA group (**p < 0.01, ****p < 0.0001) (Figure 2). Furthermore, placental tissues were collected from the AGA group (*n* = 5) and the IUGR group (*n* = 5). RNA extraction, reverse transcription, and PCR amplification were performed to evaluate the differences in Klotho mRNA expression between the two groups. The findings revealed that placental Klotho mRNA expression was significantly reduced in the IUGR group compared with the AGA group (****p < 0.0001) (Figure 3).

#### 3.2.3 IUGR placental tissue shows reduced expression of Klotho protein detected by immunofluorescence

**Figure 4:**
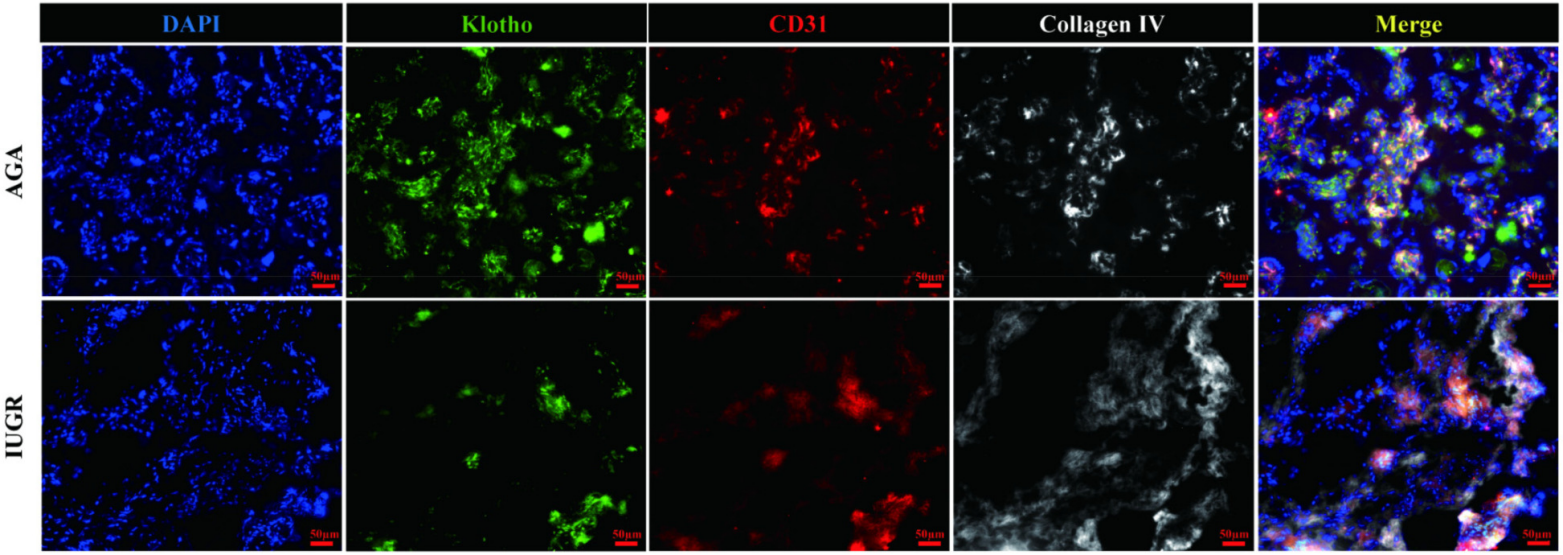
Immunofluorescence staining of Klotho (green), endothelial cells (red), extracellular matrix (gray), and nuclei (blue). The scale bar in the figure represents 50μm, magnification: 200 × times.

**Figure 5:**
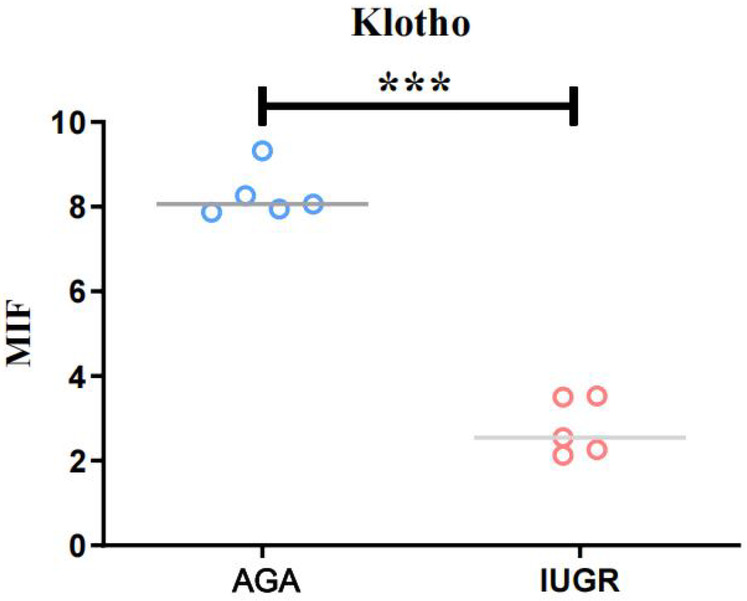
The average fluorescence intensity of Klotho protein in IUGR placentas is lower than that in AGA placentas (****: *p* < 0.0001, ***: *p* < 0.001, **: *p* < 0.01, *: *p* < 0.05, ns: not statistically significant).

The Klotho protein expression levels in placental tissue sections from the AGA group and the IUGR group were semi-quantified by immunofluorescence staining. Klotho protein was detected with a Klotho (KL) polyclonal antibody (green), endothelial cells were marked with CD31/PECAM-1 (red), extracellular matrix (ECM) was labeled with type IV collagen (gray), and nuclei were stained with DAPI (blue). The results demonstrated (Figure 4, magnification: 200× times) that Klotho protein exhibited high expression in endothelial cells, while its expression was significantly reduced in the IUGR group, coinciding with a decrease in the number of endothelial cells (scale bar: 50 μm; magnification: 200x). Fluorescence intensity was quantified by measuring the mean fluorescence intensity (MFI) using ImageJ software, with background fluorescence subtracted to ensure accurate quantification.Statistical analysis of the mean fluorescence intensity performed using Image J and GraphPad Prism 10.1.2 indicated a significant downward trend in the mean fluorescence intensity of Klotho protein (***p < 0.001) (Figure 5). An unpaired *t*-test with Welch's correction was employed to compare the meanings between two independent samples. For comparisons involving multiple groups. However, although their expression trends were spatially consistent, the reduction in Klotho was not limited to areas of endothelial loss. Therefore, while our findings suggest a relationship between endothelial cell loss and Klotho downregulation, we cannot conclude that the reduction in Klotho expression is solely attributable to a decrease in endothelial cell numbers. Other regulatory mechanisms may also be involved.

### 3.3 The expression of IGF-1 and GH protein in placenta tissue of IUGR was detected by immunofluorescence

**Figure 6:**
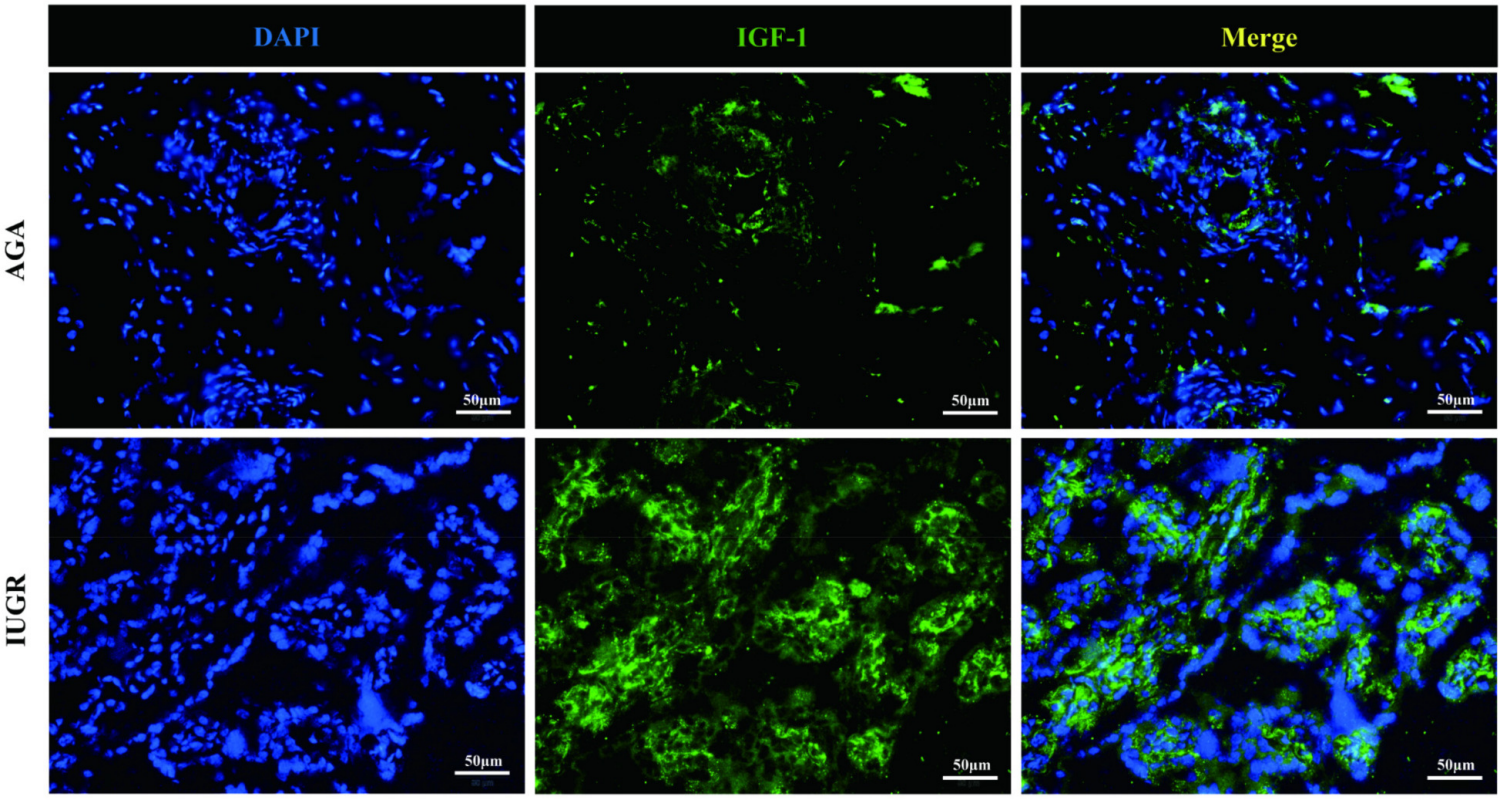
IGF-1 (green) and cell nucleus (DAPI) immunofluorescence staining. Scale bar is 50 μm, magnification: 200 × times.

**Figure 7:**
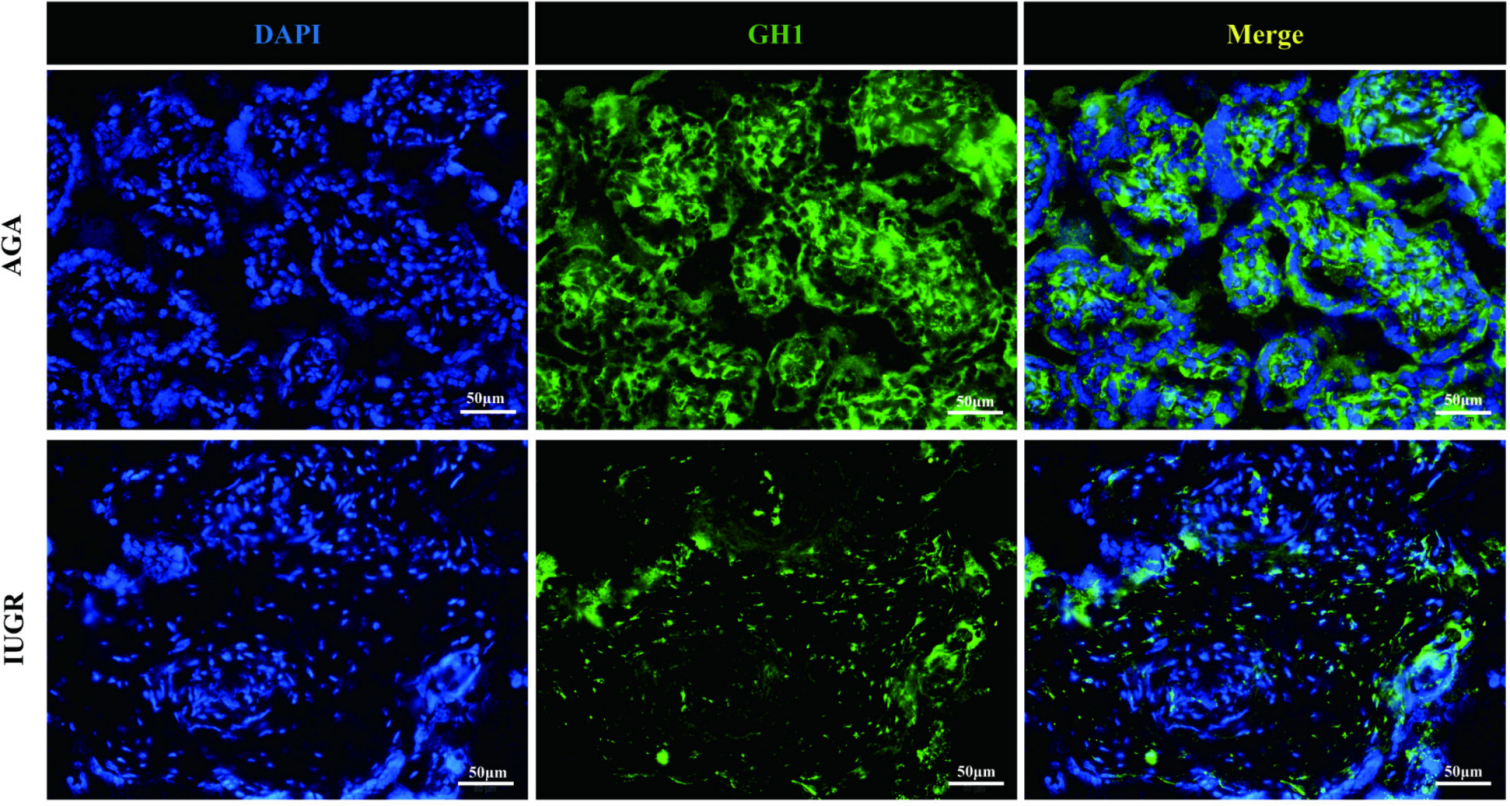
GH1 (green) and cell nucleus (DAPI) immunofluorescence staining. Scale bar is 50 μm, magnification: 200 × times.

**Figure 8:**
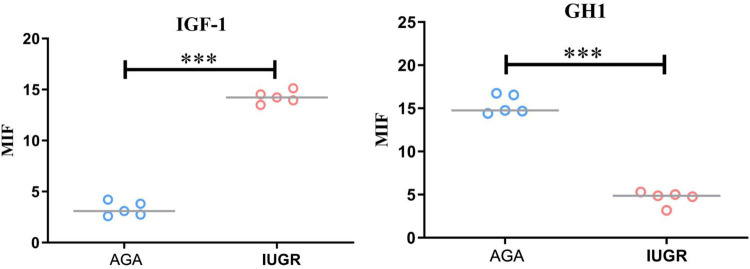
Quantitative analysis of fluorescence intensity using imageJ software. Compared with the AGA group placenta, the average fluorescence intensity of IGF-1 protein in the IUGR placenta was enhanced (***p < 0.001); the average fluorescence intensity of GH1 protein in the IUGR. placenta (*n* = 5) was decreased (***p < 0.001). (****: *p* < 0.0001, ***: *p* < 0.001, **: *p* < 0.01, *: *p* < 0.05, ns: not statistically significant).

GH1 and IGF-1 in placental samples were measured by immunofluorescence. The fluorescence intensity was quantified with ImageJ software to assess changes in protein expression associated with intrauterine growth restriction (IUGR). Immunofluorescence staining revealed IGF-1 (green) and nuclear (DAPI) fluorescence (Figure 6), with the mean fluorescence intensity of IGF-1 protein being significantly increased in the placentas of the IUGR group (*n* = 5) compared to the AGA group (*n* = 5) (***p < 0.001) (Figure 7). Similarly, GH1 (green) and nuclear (DAPI) fluorescence staining (Figures 2, 3) demonstrated that the mean fluorescence intensity of GH1 protein was significantly reduced in the placentas of the IUGR group (*n* = 5) compared to the AGA group (*n* = 5) (***p < 0.001) (Figure 8). Fluorescence intensity was quantified by measuring the mean fluorescence intensity (MFI) using ImageJ software, with background fluorescence subtracted to ensure accurate quantification. An unpaired *t*-test with Welch's correction was employed to compare the meanings between two independent samples. For comparisons involving multiple groups.

### 3.4 Changes of GH, IGF-1 and IGFBP-1 levels in venous and cord blood of IUGR pregnant women

**Figure 9:**
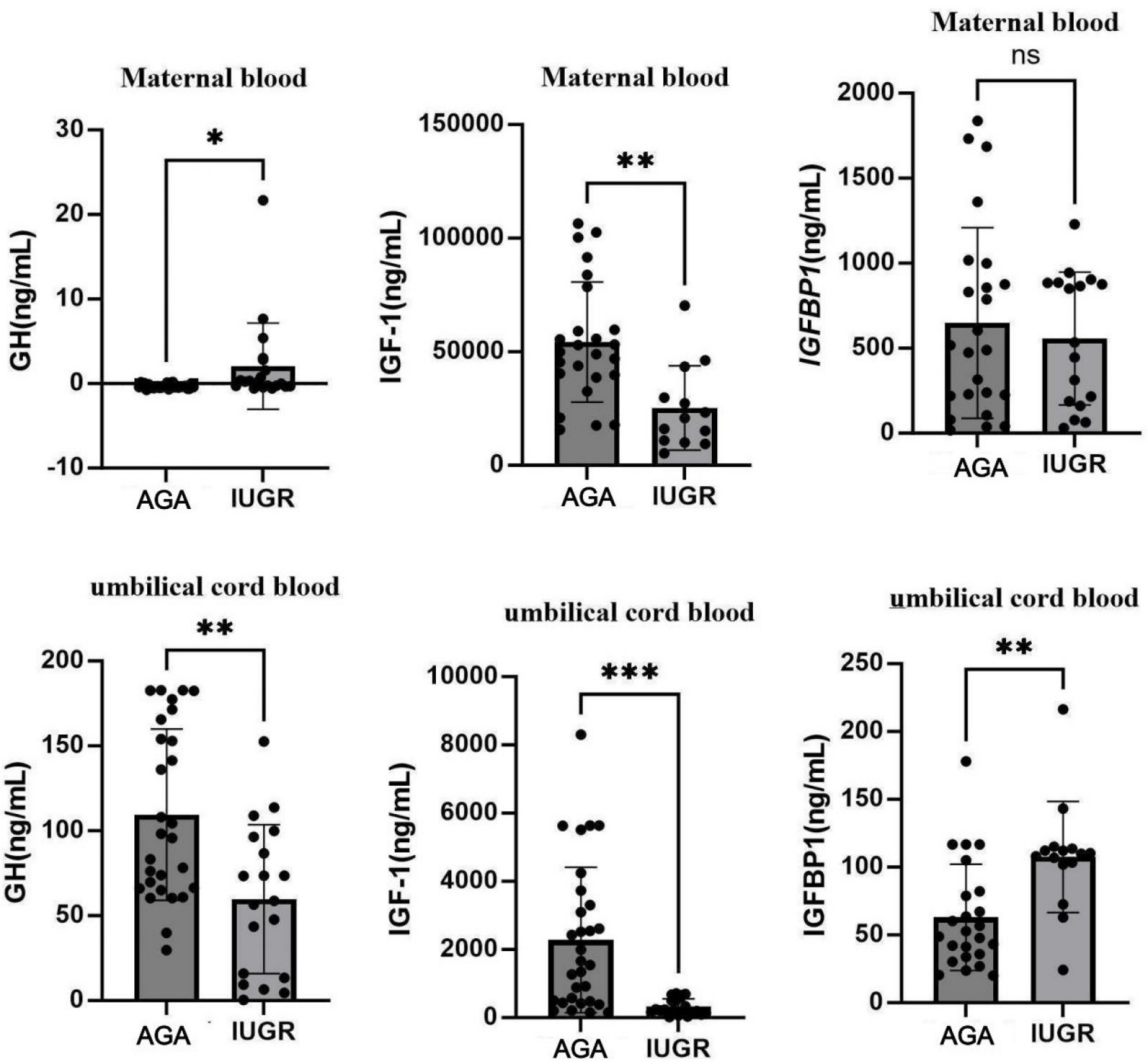
Expression changes of GH, IGF-1, and IGFBP-1 in venous blood and umbilical cord blood in AGA group (*n* = 30) and IUGR group (*n* = 22). (***: *p* < 0.001, **: *p* < 0.01, *: *p* < 0.05, ns: not statistically significant).

Serum levels of GH, IGF-1, and IGFBP-1 were determined using ELISA. The results showed that, compared with the AGA group venous blood GH levels were significantly increased in the IUGR group (*p* < 0.05), while IGF-1 levels were significantly decreased (*p* < 0.01). No significant difference was observed for IGFBP-1 levels. In the IUGR group, GH levels of umbilical cord blood were significantly reduced (*p* < 0.01), IGF-1 levels were markedly decreased (*p* < 0.001), and IGFBP-1 levels were significantly elevated (*p* < 0.01). These findings are summarized in Figures 9. An unpaired *t*-test with Welch's correction was employed to compare the meanings between two independent samples. For comparisons involving multiple groups.

## 4 Discussion

Intrauterine growth restriction (IUGR) is a common complication in pregnancy and influences morbidity and mortality at all stages of life. In IUGR fetuses, a reduced supply to the placenta causes an adaptive response that primarily transport oxygen and nutrients to vital tissues; such as the brain and heart. As a result, the available resources for muscle growth are greatly reduced, resulting in a decrease in the number of muscle fibres at birth. Intrauterine growth retardation occurs when placental supplementation does not meet the needs of the developing fetus. Infants with IUGR typically present with low birth weight and altered morphological features of allometric organ growth and are prone to perinatal complications that affect multiple body systems (18).Klotho is highly expressed in the brain, the kidney, and parathyroid and pituitary glands, but can also serve as a circulating hormone by its shedding, forming soluble klotho that can be detected in blood, cerebrospinal fluid, and urine. Mice that do not express klotho die prematurely with multiple symptoms of aging, several of them are also characteristic of decreased GH/IGF-1 axis activity (19). Studies show that the level of Klotho protein in umbilical vein blood is significantly higher than in maternal and neonatal venous blood. This suggests that Klotho protein in the placenta may participate in fetal material metabolism by being secreted into the umbilical blood, thereby affecting the birth weight of newborns (20). Another study found that Klotho mRNA and protein are highly expressed in the placental tissue of macrosomic infants, which is closely related to the birth weight of newborns (21). Does Klotho protein play a role in fetal development? Is there a correlation between Klotho protein expression and the GH/IGF-1 axis?

In our study, compared to the AGA group, the concentrations of Klotho mRNA and protein in maternal venous blood and umbilical cord blood were reduced in the IUGR group, and Klotho protein in placental tissue was also correspondingly reduced. This indicates that the reduction of Klotho protein may be one of the causes of intrauterine growth retardation. Cortex-araya Y et al. found that Klotho may be a novel and important factor affecting muscle development in IUGR (11). Research has shown that a reduction in Klotho protein leads to placental dysfunction, which in turn affects fetal growth (21).

In our study, GH expression decreased in placental tissue and umbilical cord blood in the IUGR group, but increased in maternal venous blood, whereas IGF-1 expression increased in placental tissue and decreased in maternal venous and umbilical blood. IGF-1 is an essential endocrine factor for fetal intrauterine growth, placental growth, and the maintenance of normal pregnancy. IGF-1 is mainly expressed in placental syncytiotrophoblasts and smooth villous layer cells. A small portion of IGF-1 secreted by the placenta enters the maternal and fetal circulation, while the majority primarily functions to promote placental function in an autocrine manne. In the placenta, IGF-1 binds to its receptors to promote the transfer of nutrients, such as glucose and amino acids, from the maternal side to the fetal side, thereby supporting fetal growthr (22). Some researchers have found no abnormal IGF-1 expression in placentas of the IUGR group, while others have refuted this finding. They found that IGF-1 mRNA expression levels in placentas of IUGR patients are increased, which may represent a compensatory response to abnormal fetal development. Other researchers also found that the expression of IGF-1 mRNA and IGF-1 protein in placentas of the IUGR group was lower than that in the AGA group (23). This study found that, compared to the AGA group, the expression of GH in the placenta of IUGR pregnancies decreased, while the expression of IGF-1 increased. This change promotes the transformation of placental function, facilitating the transport of more nutrients to support fetal growth, consistent with the findings of previous studies (24). This indicates that the expression changes of IGF-1 mRNA and IGF-1 protein in the placenta are positively correlated with the birth weight of newborns. IGFBP-1 binds to IGF-1, modulating its growth-promoting effects. IGFBP-1 is negatively correlated with fetal weight. In IUGR fetuses, IGF-1 decreases and IGFBP-1 reactivity increases in cord blood, indicating fetal growth restriction (22). This phenomenon is consistent with the results in the present study.

Based on the above analysis, Klotho protein expressed in placenta can promote the development of placental tissue and may participate in fetal metabolism by secreting into umbilical blood, thereby affecting the birth weight of newborns. The mechanisms by which Klotho protein affects the GH/IGF-1 axis may include: (1) Klotho protein promotes placental growth, facilitates nutrient exchange between mother and fetus, and influences fetal and placental development (24); (2) Klotho protein may affect fetal endocrine metabolism, especially by promoting pituitary secretion of GH, thereby stimulating fetal growth and subsequently impacting the GH/IGF-1 axis (25); (3) α-Klotho protein not only promotes vascular development and tissue growth but also reduces inflammation, while possessing anti-inflammatory and antioxidant effects, influencing fetal organ development (26).However, our study has some limitations. Firstly, it was a single-center study with a small sample size, and no LGA cases were included as controls. Second, although the Klotho protein analysis detected without the collection of urine samples from the pregnant women in many studies, the mechanism of whether the decreased of the Klotho protein was related to renal diseases should be further studied. Finally, we only validate the results in clinical trials. Cell experiments and animal experiments are absent. Further studies are warranted.

## 5 Conclusion

The Klotho protein is a participant in fetal intrauterine development. In cases of intrauterine growth restriction (IUGR), the expression of Klotho protein in maternal venous blood, umbilical cord blood, and placental tissue are reduced, accompanied by changes in GH, IGF-1, and IGFBP. This indicates that the expression level of Klotho protein and the balance of the GH/IGF-1 axis have an important impact on fetal intrauterine development (27, 28). Klotho protein may indirectly participate in the complex regulation of the GH/IGF-1 axis such as affecting placental function, fetal pituitar*y* axis, and fetal visceral development.Although the mechanisms need to be further researched, the current study suggests that GH/IGH-1 might have latent significance by changing Klotho protein levels for the pharmaceutical intervention of IUGR cases.

## Data Availability

The raw data supporting the conclusions of this article will be made available by the authors, without undue reservation.
